# Supplementary material for: Automatic Cerebral Hemisphere Segmentation in Rat MRI with Ischemic Lesions via Attention-based Convolutional Neural Networks
Source: Neuroinformatics. 2022 Sep 30;21(1):57–70. doi: 10.1007/s12021-022-09607-1 (PMC9931784; doi:10.1007/s12021-022-09607-1)
Supplement: Supplementary file 2 — Supplementary file2 (PDF 6.51 MB) [file 12021_2022_9607_MOESM2_ESM.pdf]

Noname manuscript No.
(will be inserted by the editor)

Automatic cerebral hemisphere segmentation in rat MRI with ischemic lesions via attention-based convolutional neural networks

Juan Miguel Valverde · Artem Shatillo · Riccardo De Feo · Jussi Tohka

Juan Miguel Valverde
AI Virtanen Institute for Molecular Sciences, University of Eastern Finland, Kuopio 70150, Finland.
E-mail: juanmiguel.valverde@uef.fi

Artem Shatillo
Charles River Discovery Services, Kuopio 70210, Finland.

Riccardo De Feo
AI Virtanen Institute for Molecular Sciences, University of Eastern Finland, Kuopio 70150, Finland.

Jussi Tohka
AI Virtanen Institute for Molecular Sciences, University of Eastern Finland, Kuopio 70150, Finland.

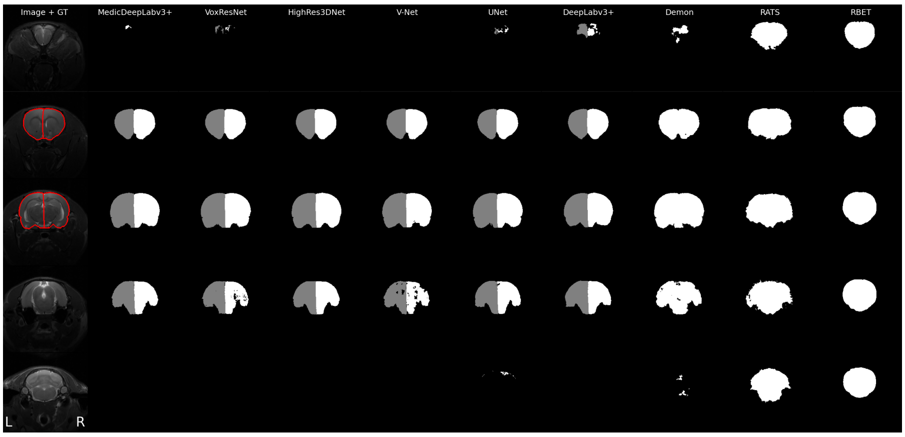

Fig. 1 T2-weighted image, ground truth and automatic segmentations of a rat from Cohort 1, 2h.

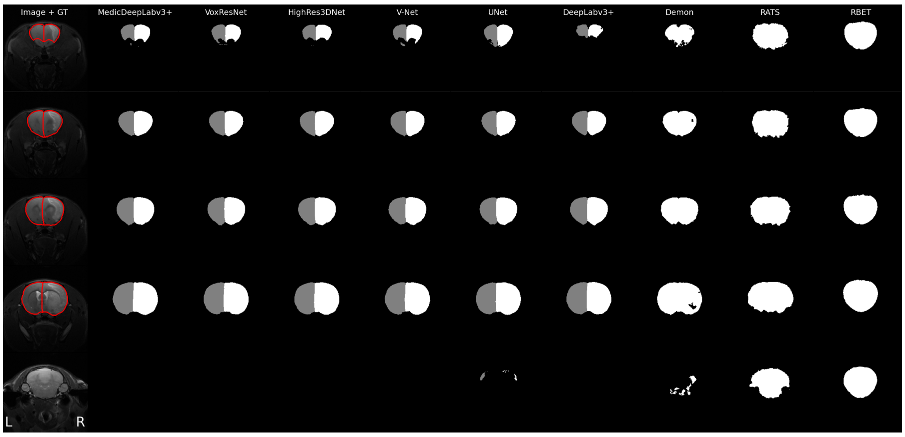

Fig. 2 T2-weighted image, ground truth and automatic segmentations of a rat from Cohort 1, 24h.

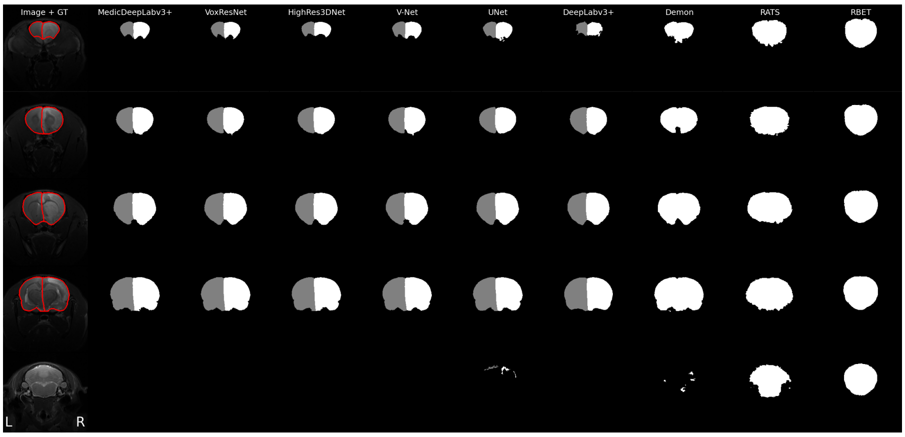

Fig. 3 T2-weighted image, ground truth and automatic segmentations of a rat from Cohort 2, 24h.

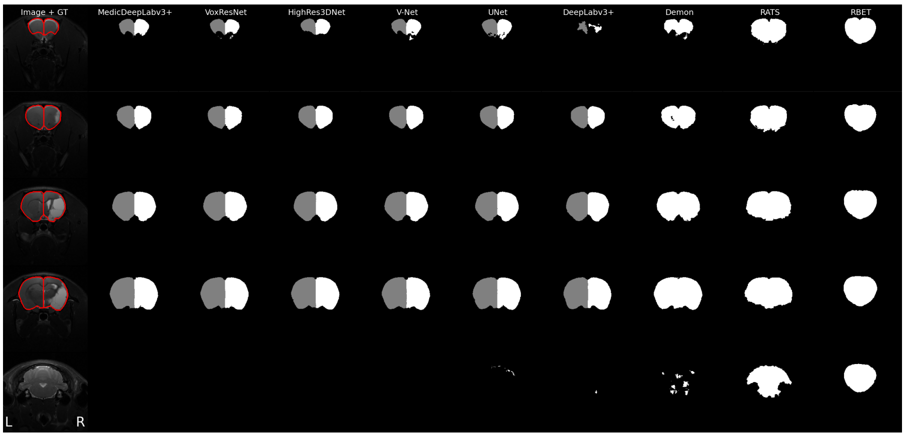

Fig. 4 T2-weighted image, ground truth and automatic segmentations of a rat from Cohort 3, D35.

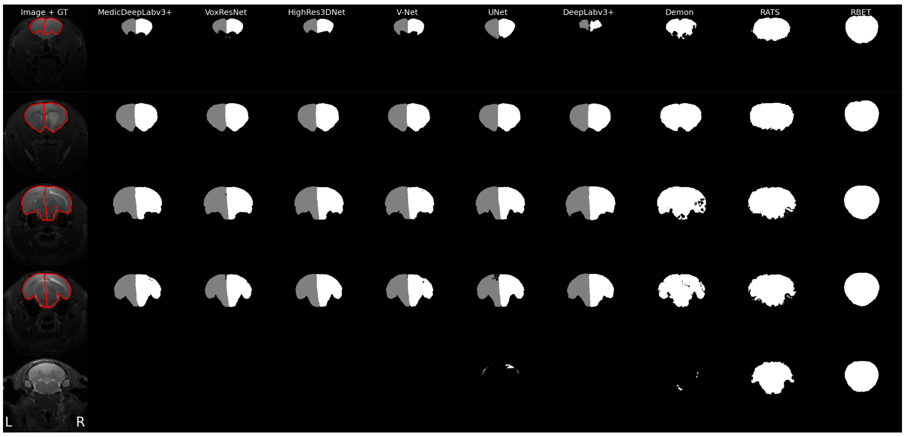

Fig. 5 T2-weighted image, ground truth and automatic segmentations of a rat from Cohort 4, 24h.

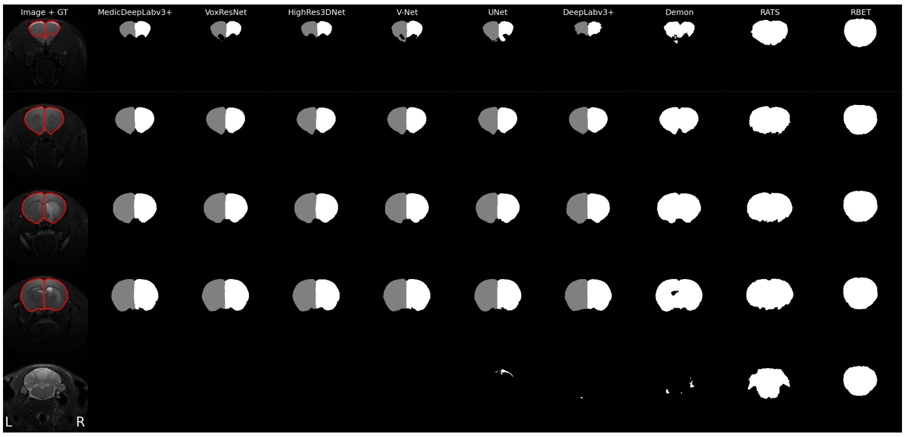

Fig. 6 T2-weighted image, ground truth and automatic segmentations of a rat from Cohort 5, 24h.

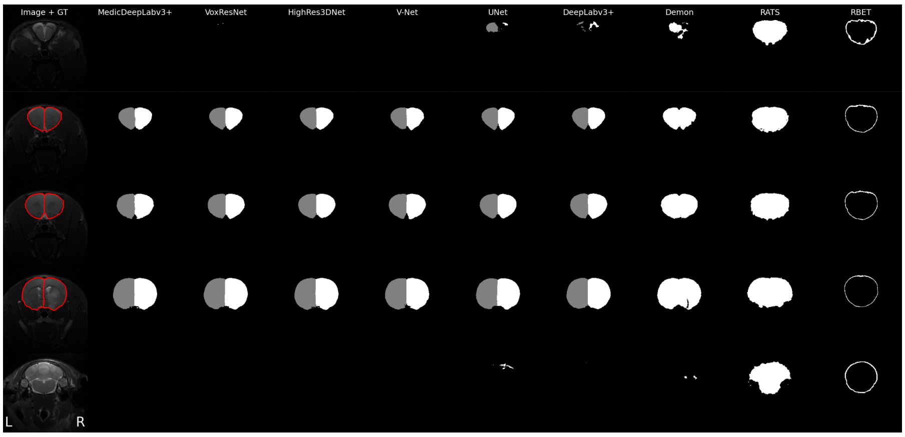

Fig. 7 T2-weighted image, ground truth and automatic segmentations of a rat from Cohort 6, D3.

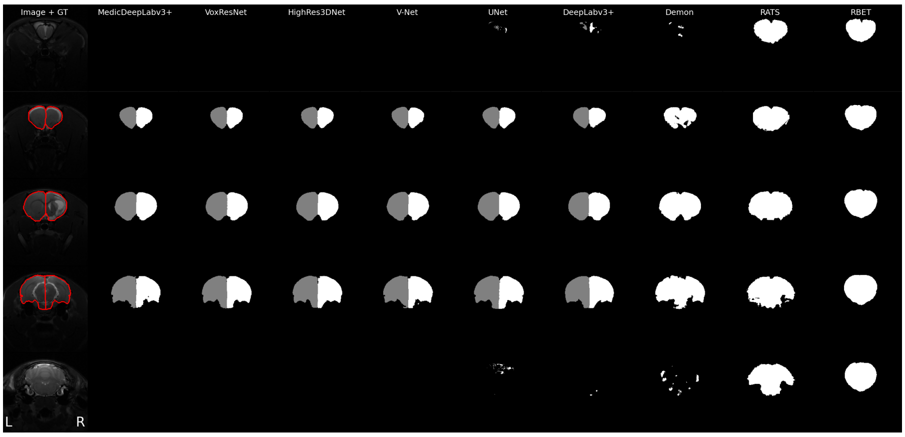

Fig. 8 T2-weighted image, ground truth and automatic segmentations of a rat from Cohort 6, D28.

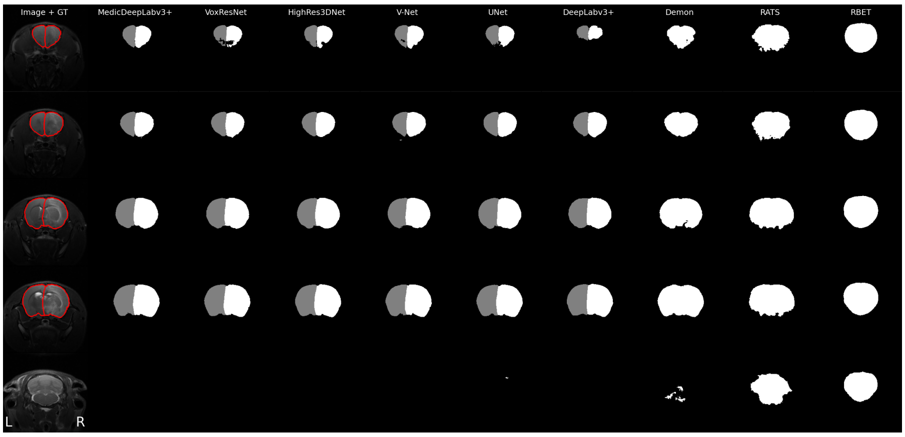

Fig. 9 T2-weighted image, ground truth and automatic segmentations of a rat from Cohort 7, D3.

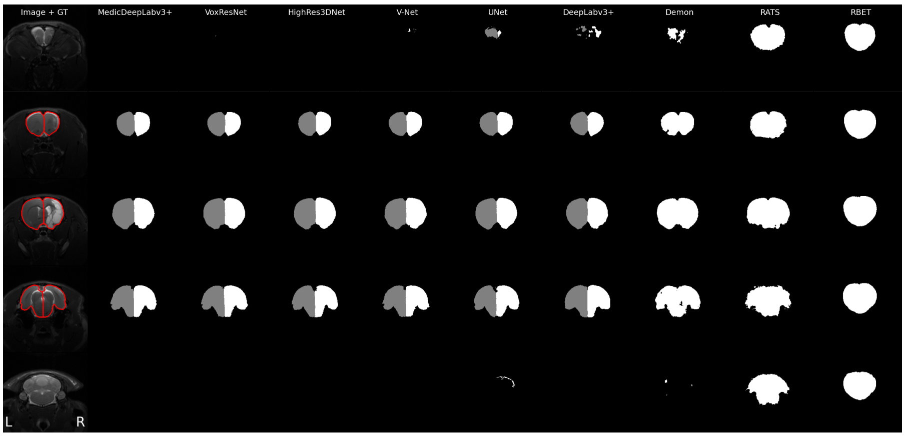

Fig. 10 T2-weighted image, ground truth and automatic segmentations of a rat from Cohort 7, D21.

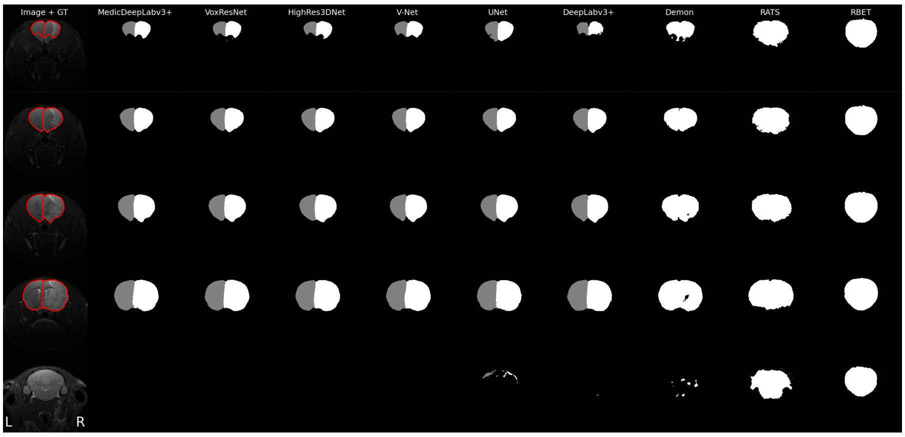

Fig. 11 T2-weighted image, ground truth and automatic segmentations of a rat from Cohort 8, 24h.

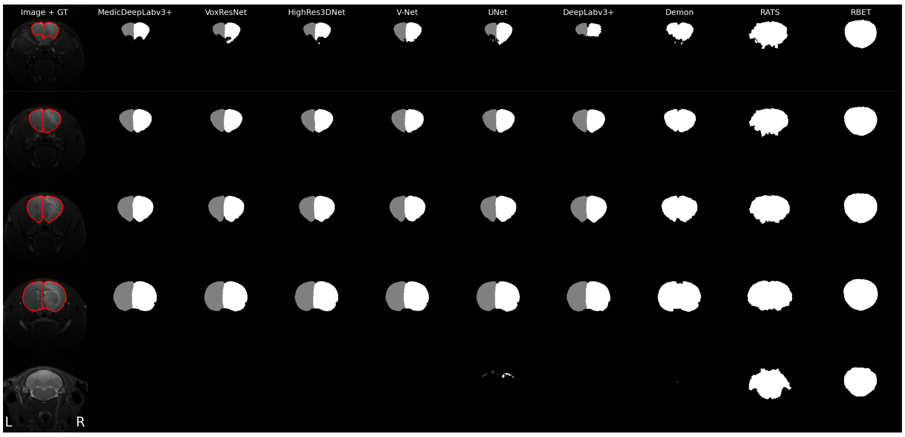

Fig. 12 T2-weighted image, ground truth and automatic segmentations of a rat from Cohort 8, D3.

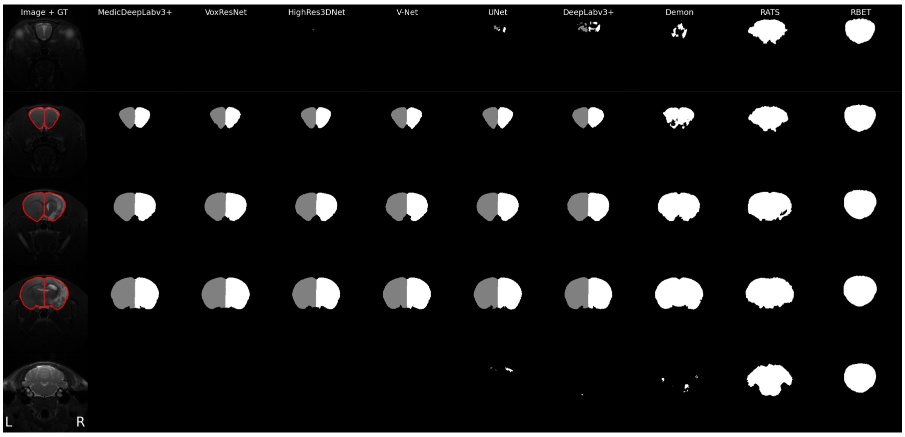

Fig. 13 T2-weighted image, ground truth and automatic segmentations of a rat from Cohort 8, D14.

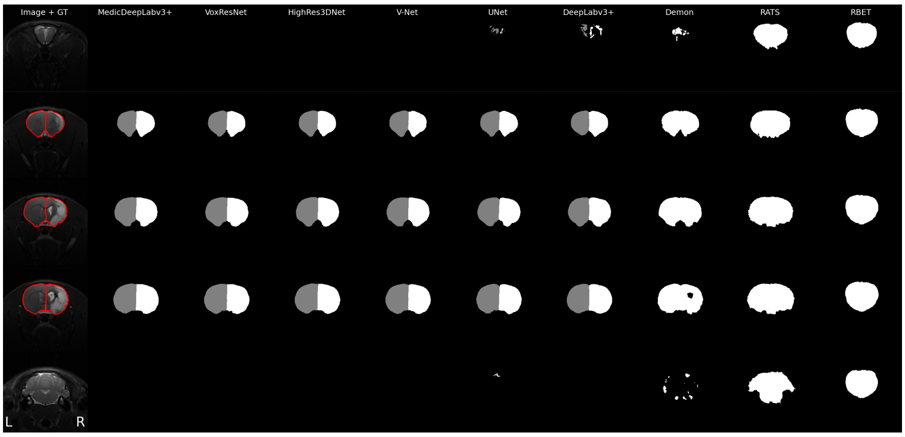

Fig. 14 T2-weighted image, ground truth and automatic segmentations of a rat from Cohort 8, D28.

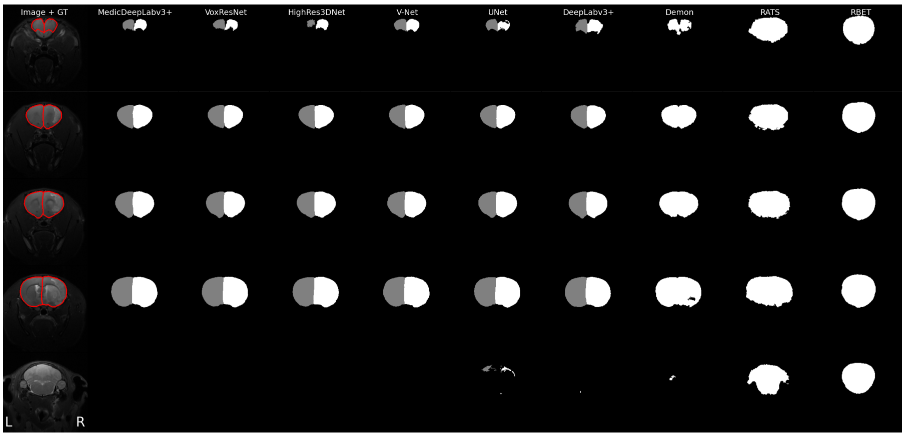

Fig. 15 T2-weighted image, ground truth and automatic segmentations of a rat from Cohort 9, 24h.

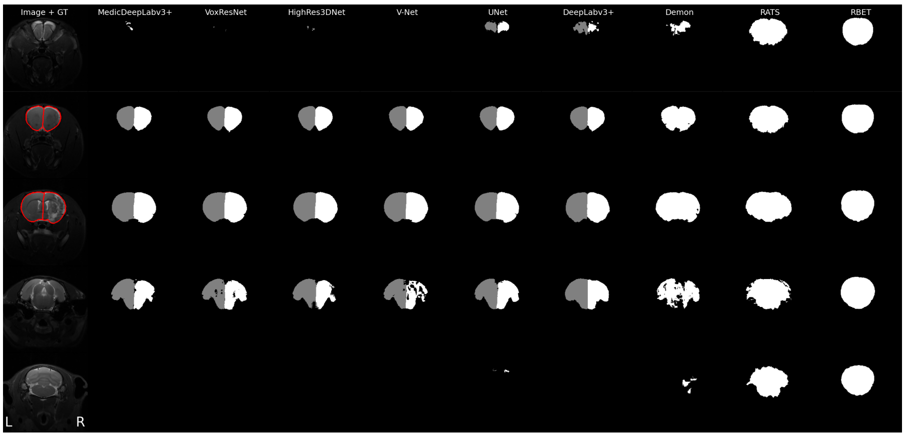

Fig. 16 T2-weighted image, ground truth and automatic segmentations of a rat from Cohort 10, D7.

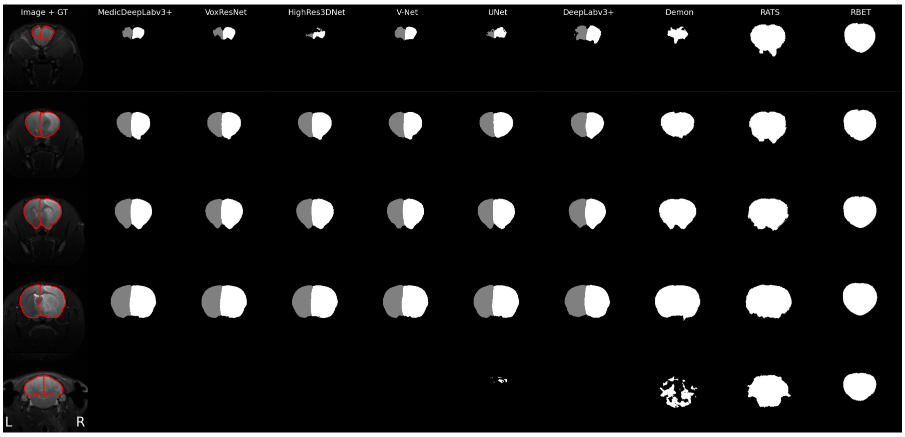

Fig. 17 T2-weighted image, ground truth and automatic segmentations of a rat from Cohort 11, 24h.
