# Supplementary material for: Automatic Cerebral Hemisphere Segmentation in Rat MRI with Ischemic Lesions via Attention-based Convolutional Neural Networks
Source: Neuroinformatics. 2022 Sep 30;21(1):57–70. doi: 10.1007/s12021-022-09607-1 (PMC9931784; doi:10.1007/s12021-022-09607-1)
Supplement: Supplementary file 3 — Supplementary file3 (PDF 90.7 KB) [file 12021_2022_9607_MOESM3_ESM.pdf]

Noname manuscript No.
(will be inserted by the editor)

Automatic cerebral hemisphere segmentation in rat MRI with ischemic lesions via attention-based convolutional neural networks

Juan Miguel Valverde · Artem Shatillo · Riccardo De Feo · Jussi Tohka

Juan Miguel Valverde
AI Virtanen Institute for Molecular Sciences, University of Eastern Finland, Kuopio 70150, Finland.
E-mail: juanmiguel.valverde@uef.fi

Artem Shatillo
Charles River Discovery Services, Kuopio 70210, Finland.

Riccardo De Feo
AI Virtanen Institute for Molecular Sciences, University of Eastern Finland, Kuopio 70150, Finland.

Jussi Tohka
AI Virtanen Institute for Molecular Sciences, University of Eastern Finland, Kuopio 70150, Finland.

Table 1 Comparison between multiple versions of MedicDeepLabv3+ with different capacity. Columns: number of initial filters, trainable ConvNet parameters (in millions), optimization time for 300 epochs in our workstation in hours, maximum GPU memory required during training and evaluation, Dice and HD in the brain mask (mean ± std). Bold: default configuration.

Filters	Parameters	Time (h)	Mem. (train)	Mem. (eval)	Dice	HD
**32**	**79.1M**	**16.2**	**8857 MiB**	**2935 MiB**	**0.952 ± 0.04**	1.856 ± 0.91
28	60.7M	14.4	7571 MiB	2617 MiB	0.950 ± 0.04	1.792 ± 0.95
24	44.7M	12.1	6545 MiB	2319 MiB	0.950 ± 0.04	1.759 ± 1.01
20	31.1M	10.3	5619 MiB	2007 MiB	0.950 ± 0.04	1.769 ± 0.98
16	20.0M	7.8	4577 MiB	1717 MiB	0.949 ± 0.04	1.707 ± 0.97
12	11.3M	6.2	3531 MiB	1421 MiB	0.948 ± 0.04	1.747 ± 1.01
8	5.1M	4.5	2503 MiB	1121 MiB	0.947 ± 0.04	**1.694 ± 0.93**
